# Case Report: Tislelizumab combined with bevacizumab in the treatment of primary malignant pericardial mesothelioma

**DOI:** 10.3389/fonc.2026.1848097

**Published:** 2026-06-16

**Authors:** Zijuan Liu, Yating Yan, Caihong Jiang

**Affiliations:** Affiliated Ordos Clinical College of Inner Mongolia Medical University, Ordos Central Hospital, Ordos, Inner Mongolia, China

**Keywords:** primary malignant pericardial mesothelioma, tislelizumab, bevacizumab, immune checkpoint inhibitors, vascular endothelial growth factor inhibitors, case report

## Abstract

**Background:**

Primary malignant pericardial mesothelioma (PMPM) is an exceedingly rare and highly malignant tumor that originates in the serosal layer of the pericardium. Currently, there are no specific guidelines for diagnosis and treatment in China and international countries for this disease, so treatment experiences from previous clinical cases are very important in the management of this disease.

**Case summary:**

A case involving grade IV bone marrow suppression following pemetrexed in combination with platinum-based chemotherapy has been documented. Subsequently, the patient underwent treatment with tislelizumab and bevacizumab (an immune checkpoint inhibitor combined with a vascular endothelial growth factor inhibitor). After 29.4 months of this treatment, the patient experienced significant relief from symptoms of chest tightness and breathlessness, while the primary tumor lesion remained stable. Imaging assessments indicated stable disease (SD). Throughout the treatment course, no severe adverse reactions resulting in mortality were observed, with only grade II bone marrow suppression and intermittent dry, scaly, and peeling skin on the feet reported. Symptoms improved following symptomatic and supportive interventions.

**Conclusion:**

This case report provides suggestive information for the application of tislelizumab combined with bevacizumab in the treatment of pericardial mesothelioma patients, especially those who are unable to tolerate the adverse effects associated with pemetrexed and platinum-based chemotherapy. The limitation is that only one patient is included, the number of cases is small, and there may be uniqueness of individual cases. Consequently, it remains uncertain whether this combination therapy is applicable to all patients with pericardial mesothelioma, nor can we ascertain whether other immune checkpoint inhibitor (ICIs) and vascular endothelial growth factor inhibitors (VEGF inhibitors) exhibit similar efficacy in this context.

## Introduction

Primary malignant pericardial mesothelioma (PMPM) is a highly malignant tumor originating from pericardium serosum with a prevalence rate of less than 0.002%. There are many risk factors for this disease, including exposure to asbestos, radiation exposure and possible viral or tuberculosis infection. Clinical manifestations include constrictive pericarditis, pericardial tamponade, and heart failure. Malignant pericardial mesothelioma is clinically rare with a poor prognosis and an average survival time of 6–10 months. In the absence of effective treatments, the treatment options for PPM are mainly derived from diffuse pleural mesothelioma (DPM), which is more frequently studied. Most patients with pericardial mesothelioma receive platinum-based chemotherapy to improve their quality of life and prolong their survival, except for some who undergo surgery. The patient reported in this case had a survival of 2.76 years, whereas previous studies of the disease found a mean survival of 6–10 months and a maximum survival of 2 years. Consider the relationship between the prolonged survival period of patients and the treatment of tislelizumab combined with bevacizumab. This has not been discussed in other cases.

## Case description

The patient is a 50-year-old female. She underwent atrial septal defect occlusion surgery in 2020,exhibited a trace amount of pericardial effusion upon echocardiographic re-examination. Subsequently, the patient experienced discomfort characterized by chest tightness, shortness of breath, and palpitations after physical activity. On November 23, 2020, she visited Peking Union Medical College Hospital, where she underwent a PET-CT examination. The findings indicated multiple nodular thickening of the pericardium, with abnormally increased radioactive uptake (SUVmax 13.7), metabolic abnormalities, and a potential for malignancy. Additionally, there was an enlarged cardiac silhouette and pericardial effusion, along with several lymph nodes in the mediastinum (regions 2, 4, and 6) exhibiting slightly elevated radioactive uptake, measuring approximately 0.5 to 0.7 cm in size, with a SUVmax of 1.8 (**Figure 1**). To further investigate the pericardial lesion, the patient underwent total thoracoscopic resection of the pericardial space-occupying lesion and biopsy at the General Hospital of the People’s Liberation Army on January 26, 2021. During the procedure, pleural adhesions were noted. After their release, a small portion of the pericardial tissue was sent for histological examination. Pathological analysis revealed that the “pericardium” consisted of fibrous and fatty connective tissue, with localized small blood vessels exhibiting tumor-like hyperplasia and focal lymphocyte infiltration. Immunohistochemical results showed CK (focal +), CD3 (T cell +), CD20 (B cell +), Calretinin (focal +), CD34 (vascular endothelial +), WT-1 (focal +), TTF-1 (-), Ki-67 (3%), and Vimentin (+). Due to the unclear nature of the pericardial space-occupying lesion, no further treatment was administered.

**Figure 1:**
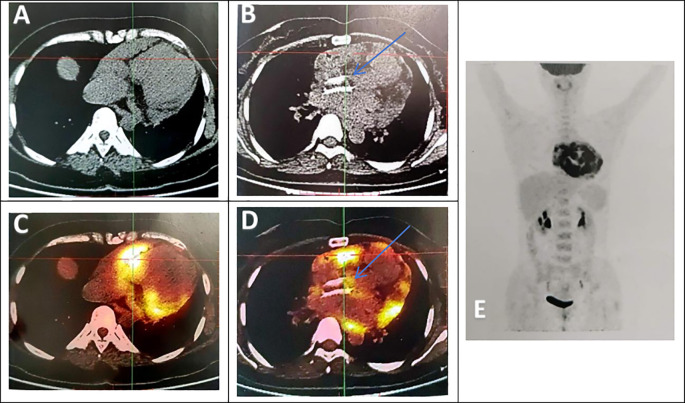
The first PET/CT in Nov.23,2020. **(A, B)** PET/CT indicated that the pericardium was significantly thickened with multiple nodules. **(C–E)** PET/CT image demonstrates a pericardial mass with avid FDG uptake, mildly elevated local metabolism,marked thickening of the pericardium with multiple nodules and increased metabolic heterogeneity. Blue arrows indicate the metal shadow after atrial septal defect occlusion surgery. PET/CT, positron emission tomography/computed tomography; FDG, fluorodeoxyglucose.

The patient presented with recurrent symptoms including chest tightness, shortness of breath, palpitations, and abdominal distension following physical activity. Physical examination of the heart and lungs showed no abnormal signs. The jugular vein was not filled or dilated, and the hepatojugular venous return sign was negative. Only symmetrical and pitting edema was observed in both lower extremities. In August 2022, a follow-up echocardiogram identified an encapsulated space-occupying lesion in the pericardial cavity, postoperative atrial septal defect occlusion, and diminished diastolic function in both ventricles, indicative of restrictive filling associated with constrictive pericarditis. To clarify the nature of the pericardial space-occupying lesion, on August 24, 2022, the patient underwent another PET-CT examination, which showed: irregular nodular thickening of the pericardium with significantly increased glucose metabolism. It is considered a malignant lesion with a small amount of pericardial effusion. (**Figure 2**) Two days later, pericardial biopsy was performed. The pathology showed that combined with immunohistochemistry, it was consistent with epithelioid malignant mesothelioma. Immunohistochemical results indicated: CKpan (+), Vimentin (+), WT - 1 (+), CR (+), D2-40 (+), Ki - 67 (15%) and CD34 (+) oven, CK5/6 (+), GATA - 3 (+) oven, P_53_ (2 +), TTF-1(-), PAX8 (-). The patient denied any history of asbestos exposure or chest radiotherapy. Her only surgical procedure was atrial septal defect closure in 2014. Primary malignant pericardial mesothelioma is challenging to diagnose due to its rarity, non-specific symptoms and signs, and diverse imaging manifestations. It must be differentiated from conditions such as malignant pericardial metastasis, direct invasion of the pericardium by pleural mesothelioma, primary pericardial sarcoma, and tuberculous pericarditis, all of which can present with recurrent chest tightness, breathlessness, abdominal distension, and edema in both lower extremities. The patient exhibited no low-grade fever, night sweats, cough, hemoptysis, or pain in the precordial area or behind the sternum. There was no prior history of tuberculosis infection or prodromal viral illness. The electrocardiogram shows no specific ST segment changes for tuberculous or viral pericarditis, even if the patient subsequently presents with arrhythmia manifestations such as atrial fibrillation (**Figure 3**). The patient’s two PET-CT examinations did not indicate diffuse pleural thickening or the presence of multiple pleural nodules and plaques. Additionally, there was no abnormal increase in radioactive uptake in the pleura. Pathological examination identified atypical mesothelial cells without the typical spindle cell configuration of sarcoma. Immunohistochemical tests confirmed positive results for Calretinin, WT-1, CK5/6, D2-40, and Vimentin, indicating the origin of mesothelial cells. The patient had no prior history of malignant tumors in the lungs or breast. The negative TTF-1 result in immunohistochemistry does not currently support a diagnosis of an epithelial-derived metastatic tumor. After analysis of the imaging and histopathology, it was thought that the primary lesion was pericardial mesothelioma. Histopathology and immunohistochemistry findings were in keeping with epithelioid-type mesothelioma.

**Figure 2:**
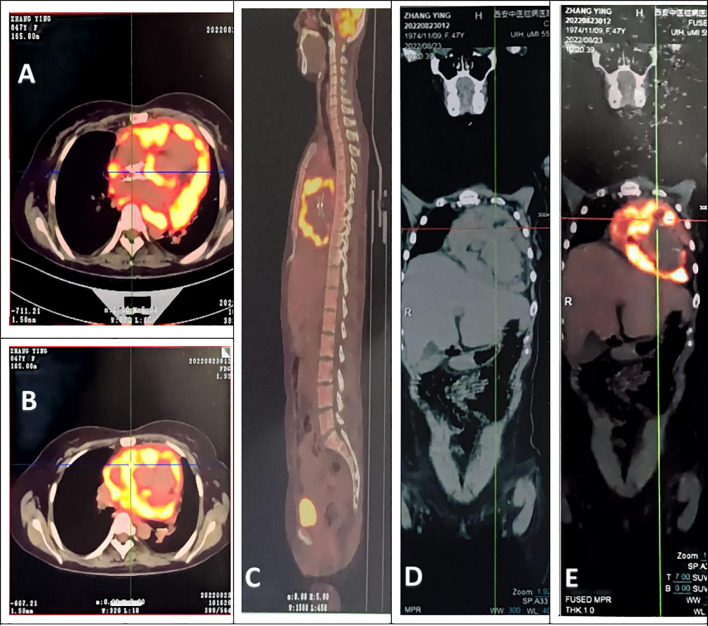
The second PET/CT in Aug. 24, 2022. **(A–E)** PET/CT shows no radionuclide visualization in the myocardium, metal density shadows can be seen in the atrial septum, irregular nodular thickening of the pericardium, and nodular and strip-shaped increase in radioactive uptake. SUVmax: 5.5-11.5. A small amount of fluid density shadow in the pericardial cavity.

**Figure 3:**
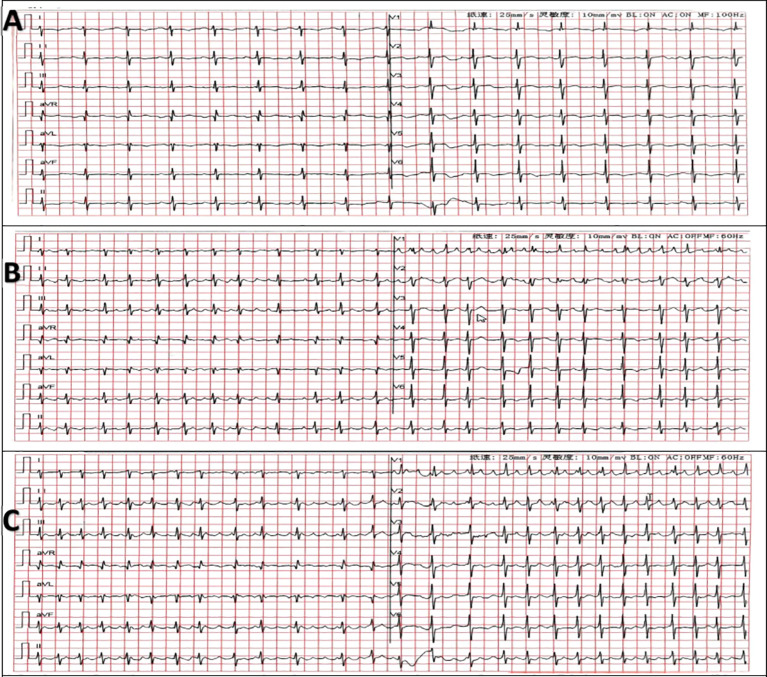
**(A)** Electrocardiogram before systemic treatment: Sinus rhythm, ST-T changes. **(B, C)** Electrocardiogram during treatment with tislelizumab combined with bevacizumab: atrial fibrillation with rapid ventricular rate, rightward deviation of the electrical axis, and ST-T changes.

The patient received a combination of tislelizumab, pemetrexed, and nedaplatin immunotherapy alongside chemotherapy for one cycle at Shaanxi Cancer Hospital on September 4, 2022. Following this treatment, the patient experienced grade IV bone marrow suppression(white blood cells:2.57×10^9^/L,neutrophils:0.77×10^9^/L, hemoglobin:99 g/L,platelets:3×10^9^/L),which was managed effectively with appropriate interventions. Given that VEGF inhibitors can enhance the anti-tumor efficacy of ICIs by modifying the tumor immune microenvironment, the combination of these agents is increasingly utilized in various solid tumors, including peritoneal mesothelioma, as evidenced by relevant case reports. Notably, using a single immune checkpoint inhibitor tends to result in milder immune-related adverse effects compared to using a combination of two. We discussed the following therapeutic regimen. The treatment plan comprised a combination of immune checkpoint inhibitors and VEGF inhibitors, scheduled from September 29, 2022, to February 26, 2025, totaling 29 cycles. The specific medication regimen included tislelizumab at 200mg and bevacizumab at 7.5mg/kg. With the patient’s weight at 60 kg, the calculated dose for bevacizumab was 450 mg. Both were administered intravenously on the first day, and then once every three weeks (i.e., 21 days as one cycle). On March 18, 2024, radiotherapy for pericardial space-occupying lesions was initiated for 10 sessions, with specific doses of 30Gy/3Gy/10f. Post-radiotherapy, the patient reported an average therapeutic effect and experienced discomfort, including pain behind the sternum, palpitations, and shortness of breath, which subsequently resolved without specific intervention. Before systemic treatment, she was 168 cm tall and 60 weighed kg. A physical examination revealed a blood pressure of 120/90 mm/Hg, heart rate 95 beats/min, and body temperature of 36.3 °C. The laboratory test results are as follows: white blood cell count is 5.26×10^9^/L, BUN/creatinine ratio is 26:1, high-sensitivity troponin T level (hstroponin T) is 0.006 ug/L, BNP is 823.9 pg/ml, cortisol is 359.5 nmol/L, thyroid stimulating hormone is 3.14 uIU/ml, FT3 and FT4 are respectively 3.69 pmol/L and 16.5 pmol/L. Echocardiography shows a mass in the pericardial cavity of the left ventricular lateral wall, decreased left ventricular function, moderate mitral valve regurgitation, slight tricuspid valve regurgitation with mild pulmonary hypertension, and bilateral pleural effusion. The radiological chest X-ray shows an enlarged shape of the heart shadow and bilateral pleural effusion. (**Figure 4**) On March 4,2025,the patient exhibited a marked deterioration in shortness of breath and abdominal distension compared to previous assessments. They experienced breathlessness with minimal exertion, and both lower extremities displayed moderate, symmetrical, pitting edema. Following diuretic therapy and enhancements in cardiac function, the symptoms alleviated. On March 11, 2025, the patient underwent chest computed tomography (CT) and echocardiography. After evaluating the treatment efficacy, which indicated progressive disease (PD), the therapeutic regimen was altered to include apalolithovolrelli in combination with anlotinib. Progression-free survival (PFS) is defined as the duration from the initial administration of tislelizumab combined with bevacizumab to the first assessment of progressive disease (PD). The received her first treatment was on September 29, 2022, and the first evaluation of PD was on March 11, 2025. Consequently, the PFS for the patient treated with tislelizumab and bevacizumab was calculated to be 894 days (i.e., 29.4 months).Following the combined treatment, the patient experienced reduced occurrences of discomfort such as chest tightness, shortness of breath, and abdominal distension compared to pre-treatment. Subsequent examinations indicated that the volume of pericardial effusion did not significantly increase and showed signs of partial absorption. Additionally, the B-type Natriuretic Peptide (BNP) levels decreased relative to pre-treatment values and remained relatively stable and low throughout the treatment course (**Figure 5**). The patient’s cardiac percussion and heart sounds were within normal limits during the physical examination. No rales were detected upon auscultation of the lungs. There has been a significant improvement in the degree and frequency of edema in both lower extremities compared to the previous assessment, indicating a relatively stable overall condition. Throughout the treatment, the patient did not experience immune-related pneumonia, pericarditis, or dysfunction of the thyroid or adrenal glands. Additionally, no cases of febrile neutropenia were observed. The only hematological adverse reactions noted were grade 2 reductions in white blood cells, neutrophils, and hemoglobin, along with grade 1 thrombocytopenia. Skin-related adverse reactions included intermittent dryness, scaling, and peeling of the feet. All aforementioned adverse reactions are amenable to symptomatic treatment. Overall, the safety profile is manageable, with no life-threatening adverse reactions, whether related to immunity or not, occurring during the treatment process.

**Figure 4:**
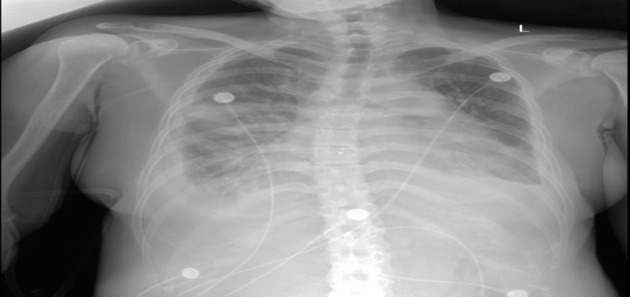
The radiological chest X-ray shows an enlarged shape of the heart shadow.

**Figure 5:**
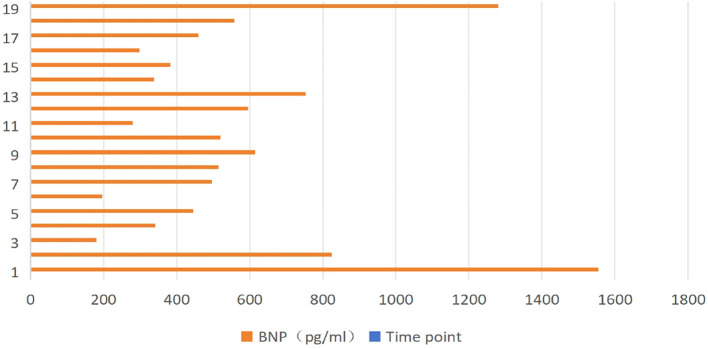
Brain natriuretic peptide response to dual tislelizumab and bevacizumab in malignant pericardial mesothelioma.The BNP level prior to tislelizumab combined with bevacizumab treatment is denoted as 1, post-treatment BNP level is denoted as 2, and the final BNP level after the last tislelizumab combined with bevacizumab treatment is denoted as 17. Numbers 2–17 signify the variations in BNP levels during the course of tislelizumab combined with bevacizumab treatment. Numbers 18 and 19 represent the BNP levels assessed after a CT evaluation of progressive disease (PD) subsequent to the modification of the treatment plan.

On April 25, 2025, the patient developed sudden breathing difficulties while undergoing apalolitovolrelli immunotherapy. Monitoring through electrocardiography revealed a decrease in blood oxygen saturation to around 85%, accompanied by elevated heart and respiratory rates. Despite prompt intervention, the patient’s vital signs stabilized. Following interdisciplinary discussions, the diagnosis of total heart failure was established, leading to the discontinuation of immunotherapy. The patient was subsequently admitted to the Intensive Care Unit for management of persistent shortness of breath, chest tightness, abdominal distension, and unrelieved edema in both lower extremities. Physical examination demonstrated enlarged cardiac boundaries on percussion, distant heart sounds on auscultation, irregular heart rate, variable intensity of the first heart sound, and scattered wet rales in both lungs. Additionally, both lower extremities exhibited moderate to severe symmetrical pitting edema. Drugs such as prednisolone, metoprolol succinate sustained-release tablets and diuretics (spironolactone, furosemide, torasemide) were administered for heart failure, but achieving control was difficult. Unfortunately, the patient subsequently died on May 29, 2025. Overall survival (OS) was defined from the date of pathologic diagnosis. The patient was pathologically diagnosed with epithelioid malignant mesothelioma on August 26,2022,and died on May 29, 2025.The OS was 1007 days (33.6 months). The treatment time points for this patient are illustrated in **Figure 6**.

**Figure 6:**
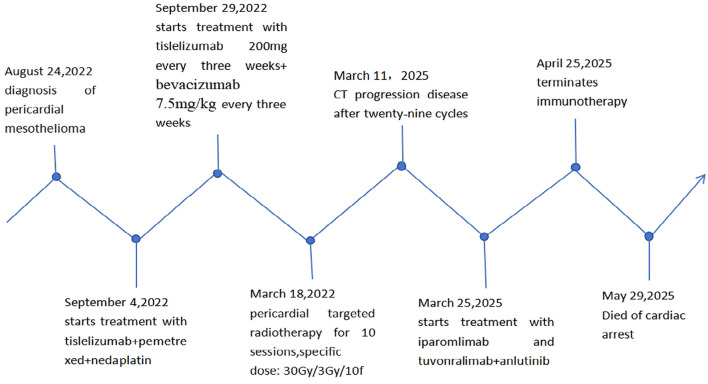
Treatment timelines.

## Discussion

Malignant mesothelioma (MM) is an uncommon, highly aggressive tumor, with an incidence rate of about 0.3 per 100,000 individuals annually (1). MM arises from serosal mesothelial cells, primarily in the pleura (88.8%), peritoneum (9.6%), thoroperitoneum (0.6%), pericardium (0.7%), and tunica vaginalis (0.2%). Pericardial-origin MM is exceptionally rare, comprising less than 1% of all mesotheliomas (2). Both the visceral and parietal layers of the pericardium can be affected, with tumor cells commonly infiltrating and spreading along the pericardium, potentially leading to widespread dissemination within the pericardial cavity. Primary malignant pericardial mesothelioma(PMPM) predominantly affects men, with a male-to-female ratio of 1.9:1. The median age of onset is 46 years, typically occurring between 50 and 70 years (3). The etiology and pathogenesis of PMPM remain unclear. While some studies link malignant pleural mesothelioma to asbestos exposure, its association with PMPM requires further investigation. Although previous literature seldom mentions asbestos exposure in PMPM patients, data from the mesothelioma registry of Lombardy, Italy, indicate that approximately 71.4% of PMPM patients have a history of asbestos exposure (4).Pericardial mesothelioma onset may be associated with viral infection, tuberculosis infection, radiation exposure, genetic susceptibility, and immune deficiency. Given the limited number of pericardial mesothelioma cases and predominantly observational study designs in current literature, a definitive conclusion regarding the causal relationship between asbestos exposure and pericardial mesothelioma cannot be established. Further investigation into the patient’s medical history revealed no documented instances of occasional or occupational asbestos exposure or a family history of tumors. According to the World Health Organization classification, pericardial mesothelioma histopathological types include epithelial, sarcoma, and biphasic types, with the epithelial type being the most prevalent (5).The patient’s pathological findings confirm an epithelioid malignant mesothelioma.

The clinical symptoms and signs of PMPM are subtle and atypical, primarily determined by the location, size, and invasiveness of the primary tumor into the pericardium. Common presentations include constrictive pericarditis, pericardial tamponade, pericardial effusion, heart failure, arrhythmias, and occasionally symptoms resembling angina pectoris. Systemic symptoms such as fever, night sweats, fatigue, weight loss, facial and limb edema, and cachexia may also occur (3). Due to its rarity, nonspecific clinical features, and ambiguous imaging characteristics, PMPM is frequently overlooked in clinical diagnosis and management, often being mistaken for pulmonary or cardiac conditions, resulting in diagnostic errors. The most prevalent misdiagnosis is tuberculous pericarditis, with other differential considerations including coronary artery disease, atrial myxoma, cardiomyopathy, lung cancer, and lymphoma. The similarity of its manifestations to those of benign pericardial diseases, coupled with an unclear etiology and the presence of other non-specific syndromes, often leads to delays in diagnosis. In many PPM cases, the time from appearance to diagnosis may last for several months or even more than a year. The duration from the onset of clinical symptoms to the pathological and immunohistochemical diagnosis of our patient was approximately one year and eight months. Therefore, in the face of recurrent or refractory pericarditis, pericardial tamponade or pericardial effusion, when there is no significant improvement in symptoms or signs after anti-inflammatory or anti-tuberculosis treatment, it is crucial to maintain a high index of suspicion for pericardial mesothelioma. Pericardial tissue pathological biopsy and immunostaining should be actively performed to confirm the diagnosis. Currently, pericardial biopsy or histopathological evaluation of surgical samples stands as the primary method for diagnosing PMPM. When combined with immunostaining and histochemical analysis, it can enhance diagnostic accuracy, particularly for epithelioid or biphasic mesothelioma. Commonly utilized positive immunohistochemical markers comprise calretinin, vimentin, Wilms tumor-1, cytokeratin 5/6, and D2-40 (6). The immunohistochemical staining results play a crucial role in the pathological diagnosis of mesothelioma. Since there are no markers with 100% sensitivity and specificity, some literatures suggest that in cases where all clinical, radiological, and histological features align, it is recommended that at least two positive markers and at least two negative markers be used as the basis for the diagnosis of epithelial mesothelioma, demonstrating a sensitivity and specificity exceeding 80% (7).In this case, Wilms tumor-1, Cytokeratin Pan, Calretinin and podoplanin/D2–40 were positive as positive markers, and TTF-1, Paired Box Gene 8 were negative as negative markers, which was one of the reasons for the diagnosis.

The non-invasive diagnosis of PMPM primarily depends on clinical manifestations and a variety of imaging techniques such as echocardiography, chest X-rays, computed tomography (CT), magnetic resonance imaging (MRI), and 18F-fluorodeoxyglucose-positron emission tomography (PET-CT) to aid in diagnosis. Cardiac ultrasound is capable of identifying pericardial effusion, pericardial thickening, or masses in the early stages, making it a viable choice for early PMPM screening (8).The patient’s cardiac ultrasounds revealed pericardial effusion and an encapsulated space-occupying lesion in the pericardial cavity. X-ray findings are influenced by the tumor’s location, extent, growth pattern, histological type, and pericardial effusion volume. These findings may include a “generalized large heart” due to significant pericardial effusion, tumor infiltration causing thickening and stiffening of the pericardium resulting in a “triangular flask shape,” as well as nodular protrusions or abnormal bulges along the heart’s border that maintain a fixed position relative to the heart silhouette. Localized pericardial mesothelioma can manifest as cardiac atrioventricular enlargement or a mediastinal tumor on CT/MRI.MRI reveals pericardial mesothelioma as an abnormal signal mass with a rapid shadow, contiguous with an arc-shaped low-signal pericardium, confirming its location in the pericardial cavity and distinguishing it from mediastinal tumors. PET-CT appears more effective than cardiac ultrasound, X-ray, CT, and MRI for diagnosing PMPM, typically manifesting as single or multiple nodular pericardial thickening with increased uptake of 18F-fluorodeoxyglucose (9). Pericardiocentesis for fluid aspiration to detect tumor cells is a diagnostic option, but its diagnostic yield is low, ranging from 10% to 30%, with a mere 20% sensitivity for detecting malignant mesenchymal cells (6). Approximately 25% to 45% of patients with PMPM exhibit metastatic lesions in locations beyond the primary pericardial site, with the lymph nodes being the most prevalent site. It has been reported that lymph node metastasis may lead to a decrease in the survival rate of PMPM patients. Additionally, PMPM frequently disseminates to adjacent large blood vessels, and extracardiac metastases can occur in the pleura, lungs, breasts, and kidneys. Some medical cases have reported metastasis to the peritoneum through lymph nodes (10).

Patients with untreated PMPM have a median overall survival of only 6 months, highlighting the critical need for effective treatment options to extend their survival. Current clinical strategies for PMPM closely resemble those for pleural mesothelioma, such as surgery (e.g., lesion excision, palliative pericardial resection, or window decompression), chemotherapy (platinum combined with pemetrexed), and targeted radiotherapy for the pericardium (11).Most patients are diagnosed with advanced-stage tumors initially, characterized by extensive myocardial infiltration and invasion of surrounding tissues, posing challenges for complete tumor resection. Forced tumor removal may inadvertently reduce patient survival. Current clinical practice often opts for palliative procedures like partial pericardial resection or fenestration to alleviate symptoms or prevent tamponade (3). Stereotactic radiotherapy has shown promising survival outcomes exceeding 3 years in some PMPM cases, albeit lacking robust evidence. Despite potential benefits, radiotherapy can lead to adverse effects such as acute pericarditis and myocarditis, with relatively high mortality rates (12).Our patient also received radiotherapy targeting the pericardium, but achieved insignificant tumor remission. Additionally, the patient reported discomfort including sternal pain, palpitations, and dyspnea. The decision to utilize radiotherapy in PMPM patients necessitates careful consideration.

Currently, systemic treatment approaches for PMPM primarily rely on case reports or extrapolation from pleural mesothelioma studies. However, there remains a notable absence of sufficient clinical randomized trials to substantiate the efficacy and safety of these regimens. McGe-hee et al. observed that among 103 PMPM patients analyzed, those who underwent platinum-based chemotherapy exhibited improved survival outcomes, irrespective of concurrent pemetrexed administration. Moreover, evidence indicates that in PMPM cases with prior tumor metastasis, the continuation of pemetrexed maintenance therapy subsequent to pemetrexed and cisplatin combination treatment may partially impede disease progression and extend survival duration (13).

ICIs are commonly referred as immunotherapy are antigens on the cancer cells, and inhibitors are the antibodies produce by the T cell defence system. By inhibiting immune checkpoints, the cancer cells become visible to be identified as abnormal cells and attack by the body’s defence system. The programmed death receptor-1 (PD-1) and programmed death receptor ligand-1 (PD-L1) inhibitors are commonly used immune checkpoint blockers for anti-cancer treatment (14).A recent phase 3 study named CheckMate 743 (15) demonstrated the efficacy of a combination of two monoclonal antibodies (nivolumab and ipilimumab) in the treatment of unresectable pleural malignant mesothelioma. This combination of checkpoint inhibitors was most effective in treating epithelioid subtypes with a durable improvement in overall survival seen. This was most notable at two years with 41% overall survival in the ipilimumab and nivolumab arm, compared with 27% for the standard platinum-based chemotherapy arm. Although some improvements have emerged in the immunotherapy of pleural mesothelioma, it remains to be seen how transferable these benefits will be to the treatment of PMPM. Some case reports show that patients with pericardial mesothelioma have seen prolonged survival or disease remission after the application of ICIs, but there are also case reports documenting disease progression after the initiation of immunotherapy. A young female patient treated with pembrolizumab in the second-line setting, followed by atezolizumab, achieved stable disease and long-term survival exceeding 4.5 years (16). In contrast, a 36-year-old male with biphasic disease who received first-line nivolumab experienced rapid disease progression (10). Furthermore, a case involving a 62-year-old female who received pembrolizumab in combination with chemotherapy resulted in a partial response and disease control for five months (17). A report concerning first-line dual immune checkpoint inhibitors in a 74-year-old male also suggests the potential of immunotherapy, although detailed outcomes were not provided (18).

Preclinical evidence indicates that angiogenesis inhibition is significant in mesothelioma, with vascular endothelial growth factor (VEGF) playing a pivotal role in its pathogenesis. Consequently, targeting VEGF may effectively enhance survival. Vascular endothelial growth factor (VEGF) pathway is functional in MPeM and VEGF inhibition results in decreased proliferation and metastasis *in vivo*. An active VEGF axis also facilitates immune evasion. VEGF inhibition, by converting an immunosuppressive tumor microenvironment to an immunopermissive one through increased infiltration of immune effector cells and better antigen presentation, can augment responses to ICI (19).Given the consistency of the histological origin of mesothelioma, pericardial mesothelioma may also exhibit elevated VEGF expression and a dependence on angiogenesis. Anti-angiogenic therapy is theoretically a potential treatment direction. Bevacizumab is a humanized anti-VEGF antibody that inhibits angiogenesis. The addition of bevacizumab to standard-of-care chemotherapy has provided a novel therapeutic option in a range of advanced cancers. Several randomized controlled trials have been conducted to investigate its efficacy in different types of cancers, including colorectal cancer, lung cancer, breast cancer, renal cell carcinoma, cervical cancer, glioblastoma, and ovarian cancer (20).We hypothesized that combining ICI and antiangiogenic therapy can have synergistic activity in PMPM.A recent meta‐analysis by Lopez‐Olivo et al. (21), which examined 23,897 cancer patients with pre-existing AD across 95 studies found that ICI therapy slightly increased the risk of IRAEs (relative risk 1.3, 95% CI 1.0-1.6) although most were mild (grade < 3), manageable with corticosteroids, and did not affect tumor response. This finding supports the cautious but feasible application of ICIs in individuals with AD under close clinical surveillance. In a case report detailing a patient with a 10-year history of idiopathic pericarditis who subsequently developed pericardial mesothelioma, treatment was administered using immune checkpoint inhibitors. After being diagnosed with mesothelioma, dual immunotherapy was applied, but the patient’s idiopathic pericarditis did not worsen. At the same time, compared with patients without immune-related diseases in the past, there was no significant difference in the occurrence and severity of immune-related adverse reactions. Finally, the article proposes that can cautiously and feasibly apply ICIs in individuals with AD under close clinical surveillance (22).In our case, after the patient made progress in treatment with tislelizumab combined with bevacizumab, she was treated with apalolitovolrelli(PD-1 and CTLA-4 dual immunotherapy) combined with anlotinib. After only one cycle of application, she stopped treatment due to severe total heart failure. From this patient, it can be observed that the probability of immune-related adverse reactions caused by the application of dual immune checkpoint inhibitors may increase compared to single immune checkpoint inhibitors. Due to the rapid progression of the disease in the later stage of the patient, it is not clear whether immune-related pericarditis has occurred, but this possibility cannot be ruled out. In fact, when we apply immune checkpoint inhibitors in patients with pericardial mesothelioma, especially dual immunotherapy we should be vigilant about immune-related adverse reactions, especially the occurrence of immune-related pericarditis. Furthermore, although the patient in this case did not undergo surgical resection of the primary pericardial mesenchymal tumor, she received a series of treatments, including immunotherapy combined with chemotherapy (tislelizumab + pemetrexed + nedaplatin), immunotherapy combined with anti-angiogenesis (tislelizumab + bevacizumab), and stereotactic radiotherapy targeting the pericardium. Due to the limited number of cases involving a single patient and the potential uniqueness of each case, it is challenging to establish a causal relationship between the prolonged survival of this pericardial mesothelioma patient and the treatment with tislelizumab combined with bevacizumab. It is also not possible to infer from this case that immune checkpoint inhibitors, VEGF inhibitors, or anti-angiogenic therapy can prolong survival in pericardial mesothelioma.

## Conclusion

This case report provides suggestive information for the application of tislelizumab combined with bevacizumab in the treatment of PMPM patients, especially those who are unable to tolerate the adverse effects associated with pemetrexed and platinum-based chemotherapy. The limitation is that only one patient is included, the number of cases is small, and there may be uniqueness of individual cases. Consequently, it remains uncertain whether this combination therapy is applicable to all patients with pericardial mesothelioma, nor can we ascertain whether other ICIs and VEGF inhibitors exhibit similar efficacy in this context.

## Data Availability

The original contributions presented in the study are included in the article/supplementary material. Further inquiries can be directed to the corresponding authors.
